# Transcriptome Analysis Reveals the Mechanism of Exogenous Selenium in Alleviating Cadmium Stress in Purple Flowering Stalks (*Brassica campestris* var. *purpuraria*)

**DOI:** 10.3390/ijms25031800

**Published:** 2024-02-01

**Authors:** Zhi Huang, Shiling Meng, Juan Huang, Wende Zhou, Xiaoli Song, Peiyao Hao, Peigen Tang, Yihan Cao, Fen Zhang, Huanxiu Li, Yi Tang, Bo Sun

**Affiliations:** College of Horticulture, Sichuan Agricultural University, Chengdu 611130, China; huangzhi@sicau.edu.cn (Z.H.); 2022205031@sicau.edu.cn (S.M.); 2022205028@sicau.edu.cn (J.H.); 2022305064@sicau.edu.cn (W.Z.); 2021205034@sicau.edu.cn (X.S.); 202001622@sicau.edu.cn (P.H.); 202001620@sicau.edu.cn (Y.C.); lihuanxiu@sicau.edu.cn (H.L.)

**Keywords:** purple flowering stalks (*Brassica campestris* var. *purpuraria*), cadmium, selenium, photosynthetic, antioxidant enzymes, glucosinolate metabolism, cadmium-responsive genes

## Abstract

In China, cadmium (Cd) stress has a significant role in limiting the development and productivity of purple flowering stalks (*Brassica campestris* var. *purpuraria*). Exogenous selenium supplementation has been demonstrated in earlier research to mitigate the effects of Cd stress in a range of plant species; nevertheless, the physiological and molecular processes by which exogenous selenium increases vegetable shoots’ resistance to Cd stress remain unclear. Purple flowering stalks (*Brassica campestris* var. *purpuraria*) were chosen as the study subject to examine the effects of treatment with sodium selenite (Na_2_SeO_3_) on the physiology and transcriptome alterations of cadmium stress. Purple flowering stalk leaves treated with exogenous selenium had higher glutathione content, photosynthetic capacity, and antioxidant enzyme activities compared to the leaves treated with Cd stress alone. Conversely, the contents of proline, soluble proteins, soluble sugars, malondialdehyde, and intercellular CO_2_ concentration tended to decrease. Transcriptome analysis revealed that 2643 differentially expressed genes (DEGs) were implicated in the response of exogenous selenium treatment to Cd stress. The metabolic pathways associated with flavonoid production, carotenoid synthesis, glutathione metabolism, and glucosinolate biosynthesis were among those enriched in these differentially expressed genes. Furthermore, we discovered DEGs connected to the production route of glucosinolates. This work sheds fresh light on how purple flowering stalks’ tolerance to cadmium stress is improved by exogenous selenium.

## 1. Introduction

In the southern region of China, purple flowering stalks (*Brassica campestris* var. *purpuraria*) are a popular vegetable during the winter and spring months [1]. They are herbaceous plants that grow in one or two years and belong to the Brassica family of Cruciferae [2]. They are also relatively resistant to cadmium accumulation. China is the origin of vegetable shoots, which have been cultivated for a long time in Hunan, Hubei, and Sichuan. However, the high cadmium concentration of the soil in Sichuan raises the risk of Cd contamination of vegetable shoots [3].

One of the main causes of heavy metal pollution in the soil environment is cadmium. In China, hazardous metals have polluted around 2.0 × 10^7^ hm^2^ of arable land, and 2.8 × 10^5^ hm^2^ of farmland is contaminated with cadmium [4]. Due to its high toxicity, lengthy half-life, and difficulty in transformation and breakdown [5], the heavy metal element Cd poses a significant risk to soil-dwelling plant life, particularly crop growth. According to Zhang et al. [6], the primary way that Cd enters the human body is through the consumption of agricultural byproducts. However, stem and leafy vegetables have recently been identified as a dietary source of heavy metals, including Cd [7].

A plant’s toxic response to Cd buildup results in dwarfing, a regression of the green hue, and a decrease in yield [8]. After being subjected to Cd stress, watermelon [9], pepper [10], kale [11], rapeseed [12], and other vegetable crops showed a significant decrease in their chlorophyll content and displayed the phenotype of leaf chlorosis. Winter wheat under Cd stress showed a significant increase in superoxide dismutase (SOD), peroxidase (POD) activity, and ascorbic acid (ASA) content, as well as a significant decrease in ascorbate peroxidase (APX) activity [13]. Under cadmium stress, the photosynthetic parameters, chlorophyll fluorescence indicators, color difference and other indicators of wheat [14], cotton [15], and mustard [16] plants showed varying degrees of reduction, leading to a decrease in their photosynthesis. An efficient method to lessen the negative effects of cadmium stress on plant growth and development is thus desperately needed.

According to Zhang and Gladyshev [17], selenium is a trace element and is referred to as the “miracle element of life” [18]. Due to its antioxidant qualities, selenium helps plants scavenge free radicals [19]. Exogenous selenium supplementation has been shown in numerous trials to help plants cope with cadmium stress. Wu et al. [20] found that selenium supplementation in rice under cadmium stress inhibited the cadmium-induced increase in leaf and root superoxide dismutase (SOD) and leaf peroxidase (POD) activities, but it increased the inhibited catalase (CAT) activity. A study conducted on selenium application under cadmium stress in winter wheat found that selenium application reduced wheat H_2_O_2_ and malondialdehyde (MDA) content, significantly increased ascorbate peroxidase (APX) activity, and restored SOD and POD activities to control levels [21]. It also strengthened the plants’ ability to withstand environmental challenges. Because of its highly active chemical nature, selenium reacts with heavy metals to form insoluble precipitates called phytochelatins (PCs), which prevent heavy metals from being absorbed. This is the reason for the antagonistic effect between selenium and heavy metals [22]. Selenium treatment dramatically increased the content of PCs in cherry tomatoes under cadmium stress, suggesting that more cadmium ions were chelated in the vesicles and lessened the damage to the plant [23]; this was also the case in rice cadmium toxicity [24].

Side chain extension, core structure synthesis, and side chain modification are the three steps in the glucosinolate (GSL) biosynthesis pathway [25]. Plant species differ in how cadmium affects the number of glucosinolates in their plants [26,27]. MYB transcription factors (TFs) are master regulators that can either directly or indirectly stimulate GSL pathway genes. MYB34, MYB51, and MYB122, for instance, control indole glucosinolate (IGS) biosynthesis in Arabidopsis, while MYB28, MYB29, and MYB76 control aliphatic glucosinolate (AGS) biosynthesis [28]. The amount of glucosinolates in oilseed rape treated with 0, 0.5, 2, 5, and 10 mM exogenous Cd did not show any discernible trend of change [29]. Research on cadmium stress in cabbage and collard leaves revealed a reduction in both GSL categories following cadmium treatment, and when compared to control plants, a significant (*p* < 0.05) decrease in the total GSL was noted [30]. The trend of transcriptome level modifications of AGS and IGS genes in the metabolic pathway of glucosinolates in kale was consistent with the fact that the amount of aliphatic GSLs in treated leaves continued to decrease with increasing treatment time, while the content of IGS increased [31]. Glucosinolate content changes in response to cadmium stress in Arabidopsis thaliana demonstrated that cadmium treatment significantly decreased glucosinolate content in roots and leaves, with a decrease in IGS content being the primary cause of this change [32]. We postulated that the pattern of changes in Brassica vegetables in response to cadmium stress is distinct from that of other vegetable species, because glucosinolate is a significant part of the qualitative composition of Brassica vegetables.

Selenium has been shown to have a mitigating effect on Cd stress in a number of crops, including radish [33], rice [34,35,36], peanuts [5], wheat [37], maize [38], and oilseed rape [39,40]. However, relatively few studies have been done on purple flowering stalks as a plant, and it is still unclear how selenium influences the plant’s defense mechanism against Cd stress at the molecular, biochemical, and physiological levels [41]. The effects of exogenous selenium in Cd-stressed conditions on purple flowering stalks, as well as the physiological and molecular mechanisms behind selenium’s influence on Cd-stress response in purple flowering stalks, must thus be thoroughly studied. The primary goal of our research was to determine how purple flowering stalks’ physiological and molecular stress response systems are impacted by exogenous selenium. By administering 250 μmol/L of sodium selenite, we were able to successfully lessen the unpleasant effects that purple flowering stalks experienced when under cadmium stress. Using RNA sequencing (RNA-seq) technology, we examined the variations in gene expression patterns among these four treatment scenarios and the changes in the plant’s growth characteristics, photosynthetic capacity, antioxidant system, and osmotic substances. The findings offer fresh perspectives on the molecular processes by which selenium responds to cadmium stress in purple flowering stalks.

## 2. Results

### 2.1. Exogenous Selenium Treatment Alleviates Cadmium Stress and Increases Biomass in Purple Flowering Stalks

**Figure 1:**
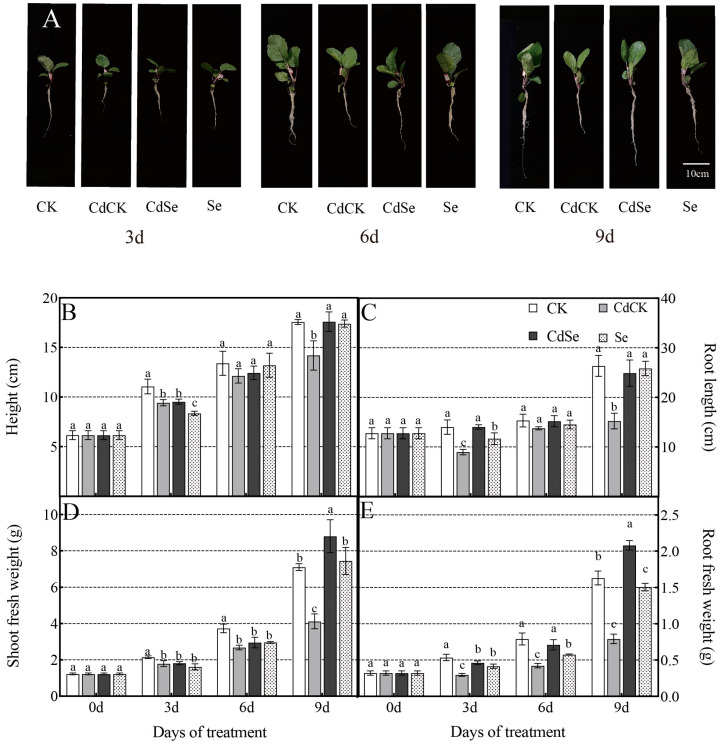
Effects of selenium treatment on the phenotype (**A**), height (**B**), root length (**C**), shoot fresh weight (**D**) and root fresh weight (**E**) of purple flowering stalks exposed to cadmium stress. The plant phenotype is 9 d after cadmium treatment. CK, distilled water plus optimal growth conditions; CdCK, distilled water plus cadmium; CdSe, selenium plus cadmium; Se, selenium plus optimal growth conditions. Each data point represents the mean of three replicate samples, and vertical bars represent the standard deviation of the mean. According to Duncan’s test, different letters indicate a statistically significant difference (*p* < 0.05).

The phenotypes of the plants under the four treatments varied after nine days of the experiment. Regularly hydrated plants (CK and Se) developed healthily. Severe yellowing and short plants were seen in CdCK plants. The plants that were exposed to less lead stress had less severe indications of leaf yellowing (Figure 1A). The physiological parameters of the leaves of exogenously Se-treated purple flowering stalk plants under Cd stress are all displayed in a table, along with their corresponding importance (Supplementary Materials).

Under cadmium stress, exogenous selenium therapy reduced the damage and enhanced the biomass of purple flowering stalk plants. The plant height, root length, shoot fresh weight, and root fresh weight of CdCK plants were all significantly reduced at the 3-day mark of cadmium stress; at the 6-day mark, CdCK plants’ shoot fresh weight and below-ground fresh weight were significantly reduced, but CdSe plants’ shoot fresh weight was significantly increased; at the 9-day mark, CdCK plants’ shoot fresh weight, root fresh weight, and shoot fresh weight were all significantly decreased, and CdSe plants’ shoot fresh weight and below-ground fresh weight were significantly increased by 23.94%, 63.82%, 1.15%, and 153.16%, respectively. This suggests that purple flowering stalks under Cd stress saw a beneficial increase in biomass as a result of exogenous selenium therapy (Figure 1B–E).

### 2.2. Exogenous Selenium Improves Photosynthetic Capacity under Cadmium Stress

**Figure 2:**
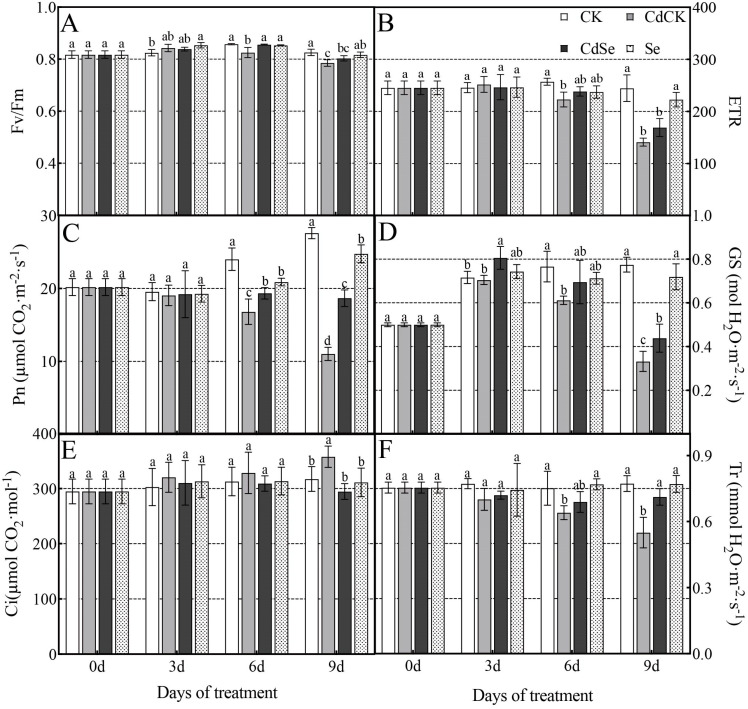
Effects of selenium treatment on photosynthetic parameters of purple flowering stalks exposed to cadmium. (**A**) Maximum photochemical efficiency (Fv/Fm); (**B**) electron transport rate (ETR); (**C**) net photosynthetic rate (Pn); (**D**) stomatal conductance (Gs); (**E**) intercellular CO_2_ concentration (Ci); (**F**) transpiration rate (Tr). According to Duncan’s test, different letters indicate a statistically significant difference (*p* < 0.05).

When exposed to cadmium stress, the Fv/Fm decreased significantly on days 6 and 9. After adding exogenous selenium, there was a tendency for an increase to occur on days 6 and 9, but only on day 6 did they show a significant increase (Figure 2A). The trends of ETR and Fv/Fm were similar, and neither of them showed a significant trend on day 3 under cadmium stress (Figure 2B).

Pn, Gs, and Tr all changed in a way that was consistent with one another (Figure 2C,D,F). Pn, Gs, and Tr all significantly decreased when exposed to cadmium stress, and these decreases were particularly noticeable at 9 days, when they decreased by 69.24, 31.95, and 29.49%, respectively. At 9 days, the decreases in Pn, Gs, and Tr of CdSe plants were slower than those of CdCK plants. Pn, Gs, and Tr in CdSe plants dropped more slowly than in CdCK plants at 9 days of Cd stress; Pn, Gs, and Tr in CdSe plants were 1.69, 1.32, and 1.29 times greater than in CdCK plants, respectively. Ci fluctuated in a manner distinct from the other three gas exchange parameters. Ci continued to rise in CdCK plants and increased by 12.72% on day 9, whereas Ci continued to deteriorate in CdSe plants and decreased by 17.64% on day 9 in comparison to Cd-stressed plants (Figure 2E).

Under Cd stress treatment, the levels of chlorophyll a, b, and total chlorophyll content all showed a similar trend of rising and then falling, which could be counteracted by CdSe plants, although the change was not statistically significant. Similarly, the levels of carotenoid content also showed a trend of rising and then falling, which could be mitigated by exogenous selenium addition, with the exception of the ninth day, which was statistically significant (Table S2).

Regarding the contrast in color under cadmium stress treatment, the brightness was indicated by the L value, which exhibited a gradually increasing trend. However, once exogenous selenium was applied, the L value showed a declining trend, with a significant decrease of 13.78% on the ninth day. After cadmium stress treatment, the a value considerably dropped on day 9, which was compatible with the phenotype of its leaf color gradually becoming greener. The yellower the fruits, the greater the absolute degree of color difference b. According to the phenotypic changes, the b values of purple flowering stalks under cadmium stress increased by 13%, 29.5%, and 84.56% on days 3, 6, and 9, respectively. After exogenous selenium application, the b values declined significantly, but the difference was not statistically significant on day 3. This suggests that exogenous selenium application had a weakening effect on the yellowness of the leaf color of purple flowering stalks under cadmium stress (Table 1).

### 2.3. Exogenous Selenium Alleviates Cadmium Stress by Increasing the Activity of Antioxidant Enzymes and Regulating Osmoregulatory Substances

**Figure 3:**
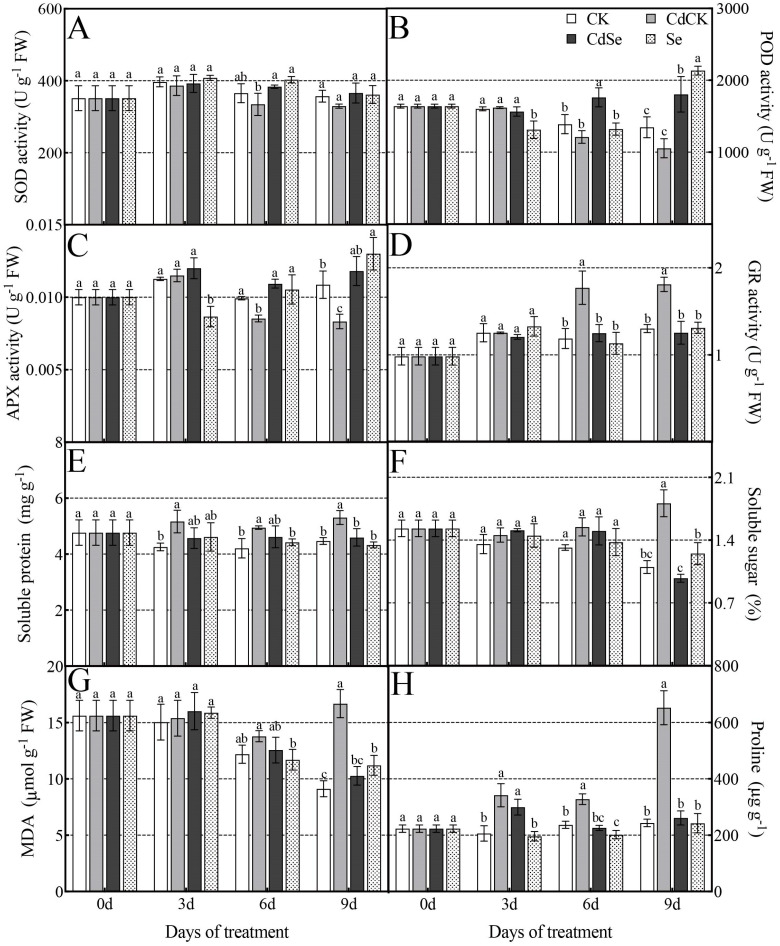
Effects of selenium treatment on the antioxidant enzyme activity and osmotic regulation in purple flowering stalks exposed to cadmium. (**A**) SOD activity; (**B**) POD activity; (**C**) APX activity; (**D**) GR activity; (**E**) soluble protein; (**F**) soluble sugar; (**G**) MDA; (**H**) proline. According to Duncan’s test, different letters indicate a statistically significant difference (*p* < 0.05).

Within nine days, cadmium treatment resulted in a sharp decline in the SOD activity of purple flowering stalks’ leaves. On days three, six, and nine, respectively, the SOD activity of the leaves under Cd treatment decreased by 2.63%, 8.5%, and 7.7% (Figure 3A). A similar trend was seen in leaf POD activity, which could be used to counteract the Cd-mediated increase in leaf POD activity to the control level, while on days six and nine, CdSe was able to significantly increase its activity (Figure 3B). In terms of APX activity, Se could effectively mitigate the Cd stress on leaves by boosting the activities of antioxidant enzymes and controlling osmoregulators. Day 9 had a notable spike in activity; Se was able to successfully mitigate the Cd stress-induced suppression of leaf APX activity, which was markedly increased on days 3, 6, and 9 of Cd exposure (Figure 3C). When Cd treatment was applied alone, leaf GR activity rose significantly (*p* < 0.05) on day 6, but the exogenous Se injection dramatically reduced GR activity by 29.43% and 30.72% on days 6 and 9 (Figure 3D).

One key sign of oxidative stress is lipid peroxidation. Furthermore, compared to CK, the soluble protein content of Cd-stressed plants treated for 3, 6, and 9 days increased significantly by 21.28%, 17.67%, and 18.85%, respectively. Moreover, exogenous selenium significantly reduced the soluble protein content of purple flowering stalks on day 9, compared to their Cd-stressed treatments, by 13.67% (Figure 3E). Plants under cadmium stress showed varying increases in soluble sugars; the changes were not statistically significant at other times, with the exception of a substantial rise on day 9 (Figure 3F). The MDA concentration of purple flowering stalks under cadmium stress was measured in order to examine the production of free radicals and the degree of membrane damage. The findings demonstrated that, in comparison to normal plants, the MDA content of cadmium-treated plants grew progressively and peaked on day 9 of the treatment at 16.68 μmol/g FW. The MDA level of cadmium-stressed purple flowering stalks treated on days 3, 6, and 9 dropped to varying degrees following the exogenous injection of selenium; the greatest notable decrease occurred on day 9, which was 38.37% (Figure 3G). Similar modifications occurred to the proline content as to MDA. The proline concentrations of plants under cadmium stress at various dates demonstrated a considerable increase compared to CK; the treatment on day 9 had the greatest proline content, measuring 652.74 μg/g. After exogenous selenium was applied, the proline contents of cadmium-stressed purple flowering stalks treated on days 3, 6, and 9 dropped by 12.38%, 31.12%, and 59.95%, respectively (Figure 3H).

### 2.4. Effect of Exogenous Selenium on Glutathione–Ascorbic Acid under Cadmium Stress

**Figure 4:**
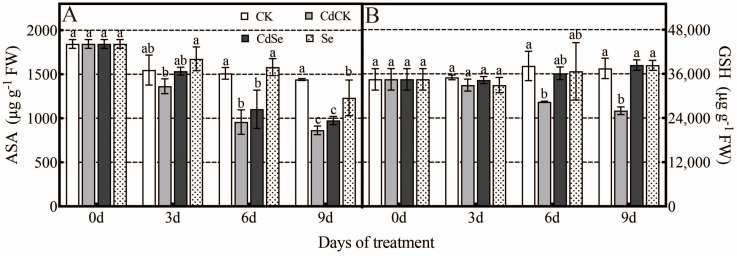
Effects of selenium treatment on the GSH-ASA cycle in purple flowering stalks exposed to cadmium. (**A**) ASA content; (**B**) GSH content. According to Duncan’s test, different letters indicate a statistically significant difference (*p* < 0.05).

When the treatment time under Cd stress increased, the amount of ASA decreased; when exogenous selenium was added, the amount increased, but there was no statistically significant difference (Figure 4A); GSH, a significant antioxidant, decreased by 25.65% and 30.65% at the 6th and 9th days of Cd stress; only on the 9th day did the GSH content of CdSe plants significantly increase, being 1.48 times higher than that under Cd stress (Figure 4B).

### 2.5. Exogenous Selenium Reduces Cd in Purple Flowering Stalks

On the third, sixth, and ninth days, the concentration of Cd in the roots was 1.52, 2.15, and 2.06 times higher than that in the shoot. The roots also had a substantially higher concentration of Cd than the shoot. Exogenous selenium supplementation considerably reduced the cadmium level in shoot CdSe in all cases, with significant reductions of 49%, 73.12%, and 54.14%, respectively. It is noteworthy that on days 6 and 9, the concentration of Cd in the CdSe-treated root increased dramatically by 71.23% and 218.21%, respectively. This suggests that once exogenous selenium was added, more Cd was immobilized in the root, restricting its transport to the shoot for mitigation. In comparison to that under cadmium stress, the translocation factor (TF) dropped by 52.72%, 84.33%, and 85.75%, in that order (Table 2).

### 2.6. RNA-Seq and Differentially Expressed Genes

**Figure 5:**
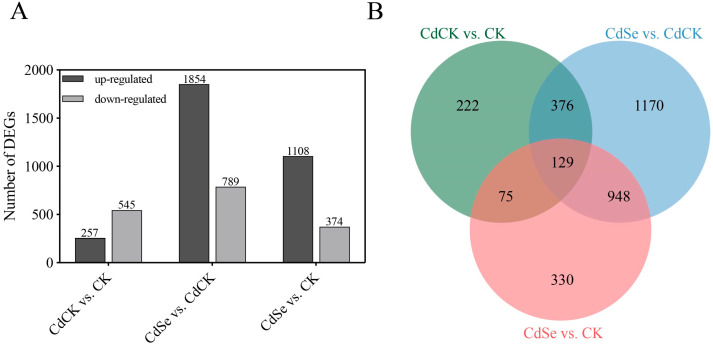
The differentially expressed genes (DEGs) in leaves of purple flowering stalks treated with cadmium or/and selenium pretreatment. (**A**) The number of upregulated and downregulated DEGs. (**B**) Venn diagram of DEGs among the comparison groups.

On day 9, twelve cDNA libraries were sequenced from four treatments (CK, CdCK, CdSe, and Se). After removing sequences that contained sequencing splice sequences, low-quality reads, sequences with high N rates, and sequences with too short a length, the high-quality sequencing data were recovered in the amount of 88.19G. Of this, the quality of clean reads that were more than Q30 was 96.79%, and the average content of GC bases in clean reads was 47.51%. The raw downstream data volume was 100.17G. The purple flowering stalks’ reference genome was retrieved from http://brassicadb.cn/#/Download/ (accessed on 19 May 2023). The percentage of similarity between each sample and the reference genome (using *Brassica rapa* cv. *Chiifu* V3.0 as the reference genome) varied from 95.97% to 96.87% (Table S3). The homogeneity of the read distribution across the genes suggested that the clean reads sequenced by RNA-seq had a low mismatch rate and were of good quality. The number of differentially expressed genes (DEGs) varied amongst the three comparisons, ranging from 2643 (CdSe vs. CdCK) to 802 (CdCK vs. CK). In CdSe compared to CdCK, there were 1854 and 789 up-regulated and down-regulated DEGs, respectively. Of the DEGs that were solely present in CdCK compared to CK, only 222 were found in CdCK, whereas 1170 were exclusively present in CdSe compared to CdCK. Furthermore, 129 DEGs in total were typically controlled across the three comparisons (Figure 5A,B).

### 2.7. Verification of RNA-Seq Data

For the purpose of quantitative real-time PCR (qRT-PCR) detection, a total of eighteen DEGs were chosen at random. According to the two approaches, almost all of these genes had identical expression patterns, indicating the reliability of the results of the RNA-seq analysis (Figure S2).

### 2.8. GO and KEGG-Enriched DEGs

**Figure 6:**
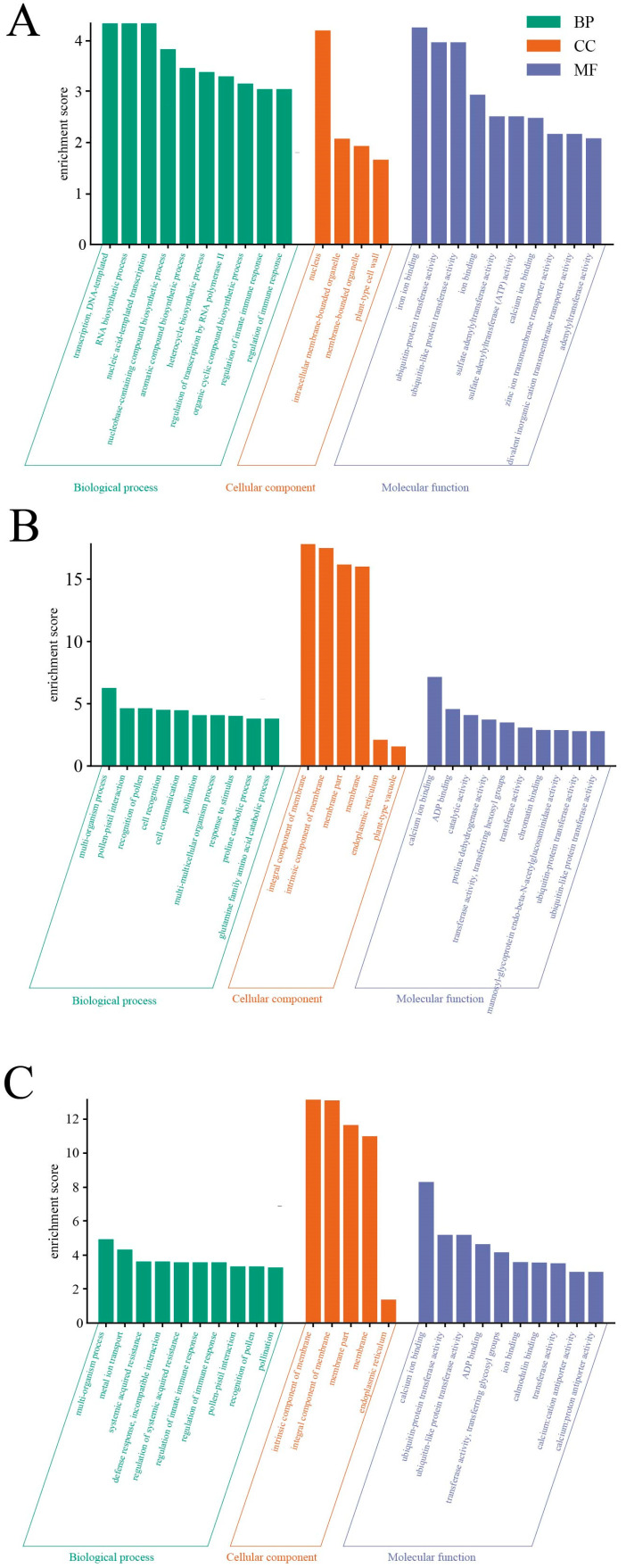
Gene ontology (GO) enrichment analysis of DEGs among comparison groups. (**A**) CK vs. CdCK; (**B**) CdSe vs. CdCK; (**C**) CdSe vs. CK.

GO analyses were performed to obtain functional annotations of the DEGs for each comparison. The top 10 enriched terms belonged to three main GO categories: molecular function (MF), cellular component (CC), and biological process (BP). The CdCK_vs._CK group was enriched with 131 GO terms. In particular, transcription, DNA-templated, RNA biosynthetic process and nucleic acid-templated transcription were significantly enriched in biological processes in the CdCK_vs._CK group; the heterocycle metabolic process and cellular aromatic compound metabolic process, respectively, were enriched by 55 genes (Figure 6A). Furthermore, 159 GO terms were enriched in the CdSe_vs._CdCK group (Figure 6B), and among the biological processes, the multi-organism process, metal ion transport, systemic acquired resistance, the defense response and incompatible interaction were significantly enriched, and the intrinsic component of the membrane, the integral component of the membrane, the membrane part of the membrane, and the membrane part were significantly enriched in the cytological component. Whereas, in the molecular function category, which was mainly enriched in calcium ion binding, were ubiquitin-protein transferase activity, the ubiquitin-like protein CdSe_vs._CK group, which was mainly enriched to the multi-organism process, the pollen–pistil interaction, the integral component of the membrane, the intrinsic component of the membrane, and the intrinsic component of the membrane, the membrane part, calcium ion binding and ADP binding (Figure 6C). Thus, selenium-relieved cadmium treatment induced the differential expression of several genes in purple flowering stalks, which involved the processes of cellular metabolism, binding, catalysis, transcriptional regulation, substance transport and signal transduction.

**Figure 7:**
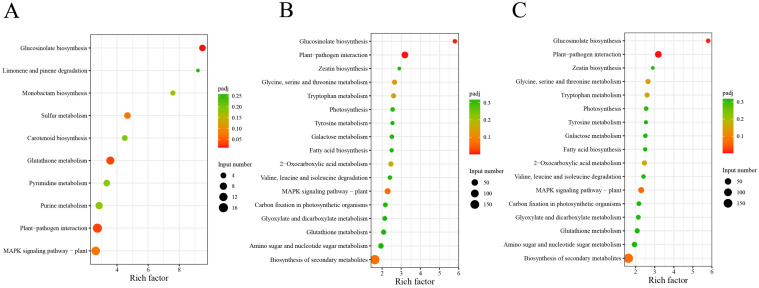
Kyoto encyclopedia of genes and genomes (KEGG) enrichment analysis of DEGs among comparison groups. (**A**) CK vs. CdCK; (**B**) CdSe vs. CdCK; (**C**) CdSe vs. CK.

KEGG enrichment pathway analyses revealed several pathways involved in exogenous selenium and the cadmium stress response. The plant–pathogen interaction had the highest number of DEGs in CdCK vs. CK, CdSe vs. CdCK, and CdSe vs. CK comparisons (16, 61, and 36, respectively). The plant–pathogen interaction, glucosinolate biosynthesis, glutathione metabolism and the MAPK signaling pathway were significantly enriched common pathways in CdCK vs. CK, CdSe vs. CdCK and CdSe vs. CK comparisons. Sulfur metabolism and carotenoid metabolism were significantly enriched in CdCK vs. CK, and photosynthesis was significantly enriched in CdSe vs. CdCK and CdSe vs. CK (Figure 7A–C).

### 2.9. Effect of Selenium Application on the Metabolism of Glucosinolate under Cadmium Exposure

Under cadmium treatment, the majority of the biosynthetic enzyme genes (BCAT4, MAM1, IPMI-L, IMDH3, CYP79F1, CYP83A1 and SOT18) and the transcription factor gene MYB28, which are involved in the manufacture of AGS, displayed active expression. Nonetheless, the majority of the genes for biosynthetic enzymes had their transcript levels markedly down-regulated by the addition of exogenous selenium.

**Figure 8:**
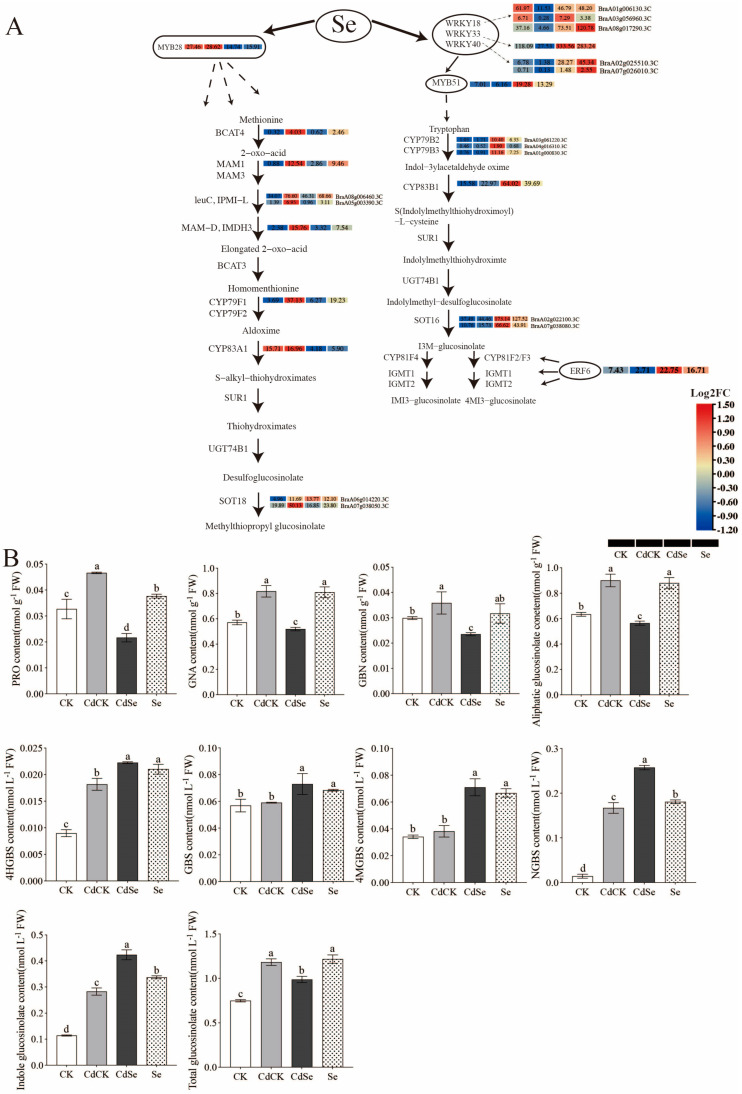
Effects of selenium treatment on purple flowering stalks on transcript profiles of the genes involved in glucosinolate metabolism pathway. (**A**) Schematic diagram of GSL biosynthetic pathway, including biosynthesis of core GSL structure and secondary modification. (**B**) Effects of selenium treatment on purple flowering stalks on glucosinolate contents. According to Duncan’s test, different letters indicate a statistically significant difference (*p* < 0.05).

WRKY transcription factors, particularly WRKY18, WRKY33, and WRKY40, have significant regulatory functions in the glucosinolate biosynthetic pathway. By binding to MYB51, they have the ability to directly control the metabolism of IGS. IGS responds to Cd exposure differently than AGS does. At Cd levels, there was an up-regulation of the expression of numerous important biosynthetic enzyme genes (CYP79B2, CYP79B3, CYP83B1 and SOT16), including the transcription factor gene MYB51. But the addition of exogenous selenium also markedly increased their transcriptome levels (Figure 8A).

We further examined the content of GSLs. The AGS content was significantly increased under Cd exposure, and the addition of exogenous selenium caused a significant decrease in its content. Combined with the transcriptome data, it was found that the significant rise in AGS after the exogenous selenium addition was caused by reduced biosynthesis and enhanced degradation. We also determined the content of PRO (progoitrin)\GNA (gluconapin)\GBN (glucobrassicanapin) in AGS, which all showed the same trend. IGS was significantly increased by exposure to Cd, and the addition of exogenous selenium resulted in a sustained and significant increase in the content of IGS. According to the transcriptome data, the increased biosynthesis and attenuated degradation in the IGS pathway caused by exposure to Cd could explain the increase in IGS content. The addition of exogenous selenium resulted in the continued activation of biosynthetic genes and the continued repression of degradation genes, leading to a continued significant increase in IGS content. We also determined the content of 4HGBS (4-hydroxyglucobrassicin)\GBS (glucobrassicin)\4MGBS (4-methoxyglucobrassicin)\NGBS (neoglucobrassicin) in IGS, and all showed the same trend. The content of the total glucosinolates increased significantly under cadmium stress, and the application of exogenous selenium led to a significant decrease in their content, which was similar to the trend of the content of AGS, indicating that AGS accounted for the majority of the content in the exogenous selenium alleviation of cadmium stress in purple flowering stalks (Figure 8B).

### 2.10. Selenium Application Responsive Genes under Cadmium Stress

The expression of almost 75% (21/28) of the DEGs implicated in the glutathione metabolic pathway was down-regulated in response to cadmium exposure. The expression of ribonucleoside-diphosphate reductase small chain C (RRM2) (brp:103854270 and brp:103875249) and glutathione S-transferase (GST) (brp:103835969, brp:103840683, brp:103831538, brp:103831540, brp:103864970) was significantly down-regulated by cadmium stress; the application of exogenous selenium produced opposite expression patterns. Under cadmium stress, there was a considerable down-regulation of several genes related to carotenoid metabolism, including abscisic acid 8′-hydroxylase (CYP707A) (brp:103839129, brp:103839129, brp:103861043). However, the down-regulation of these genes was reversed by exogenous selenium treatment. In flavonoid biosynthesis, exogenous selenium addition demonstrated a negative connection pattern with gene expression under cadmium stress, and cadmium stress dramatically down-regulated 100% (3/3) of the DEGs implicated in the flavonoid biosynthesis pathway.

**Figure 9:**
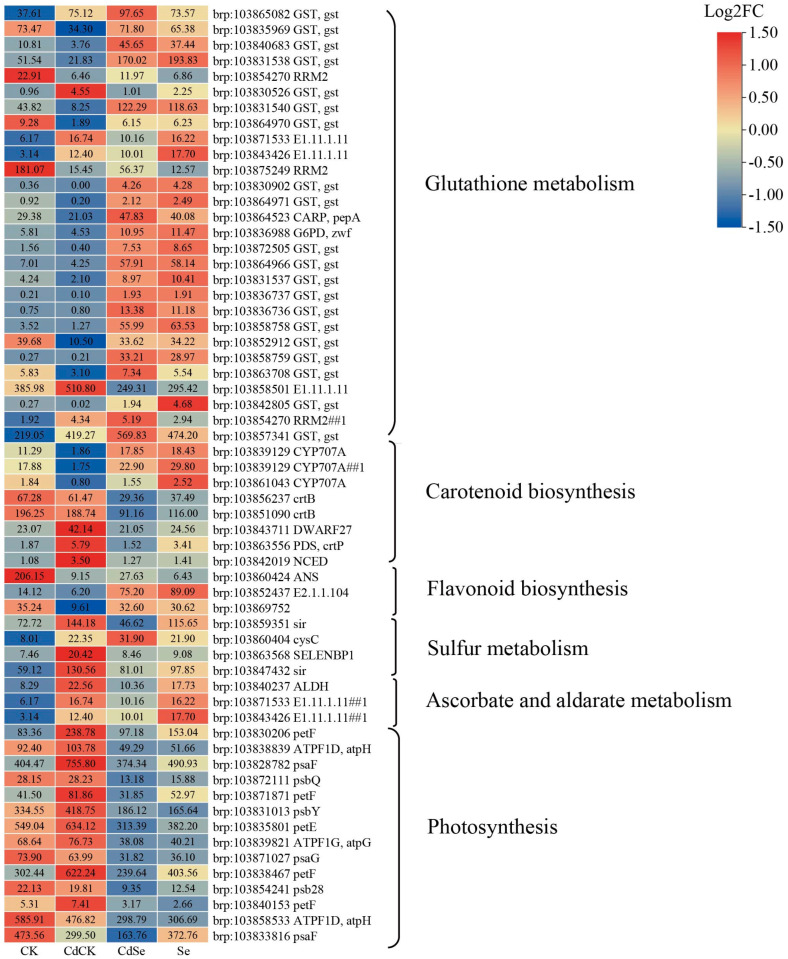
Heatmap of DEGs in KEGG pathway enrichment. Glutathione metabolism, carotenoid biosynthesis, flavonoid biosynthesis, sulfur metabolism, ascorbate and aldarate metabolism, photosynthesis.

Exogenous selenium administration affected the control of sulfur metabolism, ascorbic acid and adipic acid metabolism, and photosynthesis under cadmium stress conditions in addition to the cadmium stress-responsive genes in the aforementioned pathways. Sulfite reductase (sir) (brp:103859351 and brp:103847432), adenylylsulfate kinase (cysC) (brp:103860404), and methanethiol oxidase (SELENBP1) (brp:103863568) were all significantly up-regulated by cadmium stress. Furthermore, it is interesting to note that after exogenous selenium was added, the expression of cysC (brp:103860404) remained elevated, while the expression of the other genes reversed. Exogenous selenium reversed the considerable up-regulation of aldehyde dehydrogenase (ALDH) (brp:103840237) and L-ascorbate peroxidase (brp:103871533 and brp:103843426) expression in the ascorbate and adenylylsulfate metabolic pathways. More than 71.43% (10/14) of the DEGs involved in the photosynthesis pathway were up-regulated by cadmium stress. These included four ferredoxins (petF) (brp:103830206, brp:103871871, brp:103838467 and brp:103840153), two F-type H+-transporting ATPases (brp:103838839 and brp:103839821), two photosystem II proteins (brp:103872111 and brp:103831013), one photosystem I subunit (brp:103828782), and one plastocyanin (brp:103835801). This up-regulation was reversed when selenium was added (Figure 9).

## 3. Discussion

**Figure 10:**
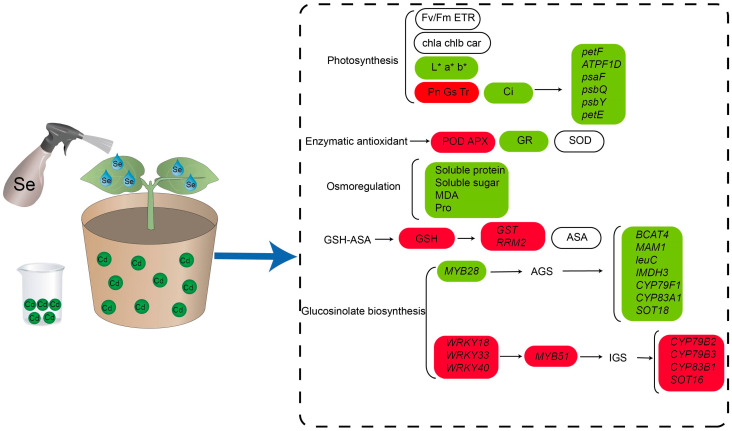
Physiological and molecular regulatory mechanisms by which exogenous selenium application enhances the cadmium resistance of purple flowering stalks. Red and green boxes indicate the increases and decreases in the content of substances and gene expression, and white boxes represent no significant changes in substance content and gene expression. * represents the value of L, a, b.

Over a lengthy period of evolution, plants have developed several survival mechanisms adapted to Cd stress. These mechanisms include the compartmentalization of cellular vesicle compartments [42,43] and chelation of Cd by plants themselves [44]. Cadmium (Cd) is a highly toxic heavy metal and environmental pollutant. According to Hasanuzzaman et al. [45], Se is a crucial nutritional element that significantly affects plant growth, development, photosynthesis, and stress tolerance. The concentrations used in studies on exogenous selenium alleviating cadmium poisoning are different among different species, for example, potato (3 mg/L) [46], tomato (20 mg/L) [47], *Brassica juncea* (80 µM ≈ 14 mg/L) [48], apple (150 mg/L) [49]. The concentration we used in this experiment was 250 µM (≈43 mg/L), which is between these concentrations. However, if selenium is applied at too high a concentration, it can induce toxic effects [50], and we will further study this issue in the future. The purpose of this study was to thoroughly examine how selenium treatment mitigates the mechanism of cadmium stress at the physiological and molecular levels. Purple flowering stalks were chosen as the research subject. Purple flowering stalks’ adaptive physiological responses to Cd stress were markedly enhanced by exogenous selenium treatment. These responses included modifications to the growth characteristics, photosynthetic capabilities, antioxidant system, and osmoregulatory components. Using transcriptome sequencing data, we found genes in purple flowering stalks that are responsive to Cd stress following the application of exogenous selenium. We also confirmed the molecular mechanism by which selenium reduces Cd stress in purple flowering stalks (Figure 10). Notably, this is the first study, to our knowledge, that sheds light on the processes by which exogenous selenium relieves cadmium toxicity in purple flowering stalks.

The chlorophyll molecule is the primary target of cadmium activity when it enters the plant body, because it is a very harmful metal element for plants. According to Li et al. [51], most of the time, Cd stress causes plant stomata to close and photosynthetic rates to drop. This leads to phenotypes such plant dwarfism and yellowing. The phenotype of purple flowering stalks was closer to its normal growth state in the current study due to the exogenous selenium treatment, which was found to be effective in mitigating the changes in the plants’ growth indices [52,53,54]. Exogenous selenium limits the amount of Cd that is transported from roots to leaves, protecting both structural and functional leaf tissues against Cd toxicity. Lower TF values provided evidence for this. Purple flowering stalks’ stomatal closure was directly correlated with the reduction of Pn, Gs, and Ci under Cd stress. Additionally, most photosynthesis-related genes, including petF, F-type H^+^-transporting ATPase, photosystem II protein, photosystem I subunit, and plastocyanin, were significantly up-regulated to offset the damage to the photosystem caused by Cd stress. Plants that receive exogenous selenium treatment may be able to repair the damaged chloroplast structure and sustain a higher rate of photosynthetic activity [55]. Filek [56] discovered that in oilseed rape, under cadmium stress, the application of 2 mmol/L selenium resulted in the reconstruction of chloroplast ultrastructure, reorganization of the structure of cystoid and stroma lamellae, and an increase in chloroplast size, fatty acid unsaturation, and fluidity of the cell membrane. The application of exogenous selenium to purple flowering stalks under cadmium stress resulted in a notable reduction in the decline of Fv/Fm and ETR. This suggests that a greater amount of light energy was received by PSII antenna pigments and utilized for photochemical processes, leading to an increased light energy conversion efficiency. The primary reason for the reduction in chlorophyll content in Cd-stressed purple flowering stalk plants was the increase in the activity of enzymes that degrade chlorophyll or the decrease in the synthesis of complexes called protochlorophyllide reductase and δ-aminolevulinic acid, which are involved in the biosynthesis of chlorophyll [57,58].

According to Lanza and Reis [59], selenium enhances the antioxidant defense system of plants by scavenging intracellular free radicals and increasing the activity of antioxidant enzymes and non-enzymatic antioxidants. Under the enzymatic antioxidant system, exogenous selenium treatment demonstrated an increase in the activities of SOD, POD, and APX and a decrease in GR activity. A similar phenomenon was observed by [38,60]. At the molecular level, exogenous selenium increased the transcriptome levels of most genes involved in glutathione metabolism, including glutathione S-transferase (GST) and ribonucleoside-diphosphate reductase small chain C (RRM2). This is consistent with the observation that exogenous selenium can also increase GSH levels in non-enzymatic systems. When plants are subjected to abiotic stressors, osmotic agents such as soluble sugars, soluble proteins, total phenolics, and proline control the osmotic potential inside plant tissues [61]. Exogenous selenium significantly reduced the levels of soluble protein, soluble sugar, MDA, and proline under Cd stress. Similar to purple flowering stalks, Wu [39] discovered that selenite decreased the amount of superoxide anion, hydrogen peroxide, and malondialdehyde caused by cadmium stress and delayed.

A transcriptome study of the leaves of purple flowering stalks subjected to Cd with or without selenium revealed that exogenous selenium had a considerable impact on secondary metabolites, particularly glucosinolates, which responded strongly to Cd. According to Zhao et al. [62], AGS and IGS are plant secondary metabolites that contain sulfur and nitrogen. It is interesting to note that exogenous selenium both boosted Cd-stressed IGS production and reduced Cd-induced AGS biosynthesis. In order to achieve the exogenous selenium alleviation of Cd stress and guarantee the supply of GSH under Cd stress, we hypothesized that the reduction of AGS production under exogenous selenium-mediated stress may also lower GSH consumption. The transcription factors of the WRKY family, particularly WRKY18, WRKY33, and WRKY40, significantly increased the transcriptome levels of MYB51 under Cd stress in purple flowering stalks when exposed to exogenous selenium. These factors directly regulate IGS metabolism via binding to promote the associated gene MYB51. It is interesting to note that some transcription factors, including ERF1, WRKY28, WRKY33, MYB28, MYB29, and MYB51, were also discovered in the transcriptomes of earlier radish roots under lead stress [63]. MYB51 displayed an up-regulation pattern in our investigation, whereas MYB28 displayed a down-regulation pattern. It is reasonable to assume that these differently produced stress signals are important players in plant signaling pathways triggered by Cd. Interestingly, the down-regulation of AGS biosynthesis after exogenous selenium infusion occurred prior to the activated aldoxime production. This is because the reduction in biosynthesis that happens before the formation of the activated aldoxime limits sulfur entry into the AGS pool from GSH and PAPS. This is because the depletion of the sulfur donors glutathione (GSH) and 3′-phosphoadenosine-5′-phosphate sulfate (PAPS) occurs after the formation of the activated aldoxime. Thus, it is anticipated that a decrease in AGS production will result in a decrease in GSH consumption, thereby achieving the objective of providing the exogenous selenium relief of Cd stress to guarantee GSH availability under Cd stress.

## 4. Materials and Methods

### 4.1. Test Materials and Pretreatment

The quality of purple flowering stalks was utilized in the tests for early-maturing red rape with purple flowering stalks with purple flowers that were acquired from Chengdu Toyo Seed Industry Co. Chengdu, China.

Nanjing Chemical Reagent Co. provided the test reagents, which were Cd^2+^ in the form of analytically pure CdCl_2_-2.5H_2_O and Se in the form of analytically pure Na_2_SeO_3_.

The examined seeds were from purple flowering stalks that were uniformly sized and full. The seeds were sterilized for 10 min using 2% H_2_O_2_ and then washed with ultrapure water.

### 4.2. Test Methods

Sterilized seeds of purple flowering stalks were sown in 72-well hole trays with substrate, which was made of perlite and vermiculite in a 3:1 ratio, and placed in a solar greenhouse for growth. The Hoagland solution was watered once every five days to ensure that the substrate layer was kept moist and replenished with nutrients. The experiment was carried out from January to June 2023 at Sichuan Agricultural University, Chengdu Campus (30°71′ N, 103°87′ E). The seedlings of purple flowering stalks that grew uniformly to three leaves and one heart were chosen, and they were then transferred into plastic pots with perlite and vermiculite (18 cm in diameter by 15 cm in height) and allowed to rest for three days. Next, using purple flowering stalks as test subjects, an exogenous selenium treatment was applied at the seedling stage in order to ascertain the effective dose of exogenous selenium. Four groups of 120 plants each received different doses of Na_2_SeO_3_: 0 (control), 10, 50, and 250 μmol/L. When compared to other treatment groups and control plants, purple flowering stalks plants treated with 250 μmol/L Na_2_SeO_3_ showed a significant improvement in Cd stress symptoms (Figure S1, Table S1).

Following that, four treatments—(1) CK; (2) CdCK; (3) CdSe; and (4) Se—were created using Cd-Se interactions on the seedlings of purple flowering stalks. Each treatment consisted of six pots and three replications. Selenium was treated at a concentration of 250 μmol/L and sprayed on the adaxial and abaxial surfaces of the leaves up to the level of droplets (approximately 20 mL). This was done every 2 days for a total of 5 sprays until harvest. Cadmium was treated at a concentration of 100 mL of 200 μmol/L in the form of CdCl_2_-2.5H_2_O, which was added directly to the Hoagland solution and applied every three days for a total of three applications. To reduce the effects of marginal effects, pots and planters were shifted about throughout the growth of the seedlings of purple flowering stalks. For transcriptome analysis, a number of randomly chosen seedling samples from each treatment were frozen in liquid nitrogen and kept at −80 °C on days 0, 3, 6, and 9. For physiological indicators, additional seedlings were taken and split into their roots and leaves.

### 4.3. Growth Indicator

Following the complete harvesting of the plant, the purple flowering stalks were washed with tap water and deionized water in turn. They were then immersed in 20 mmol/L EDTA-Na_2_ for 20 min and finally dried with absorbent paper. The plant height and root length were measured using vernier calipers, and the fresh weight of the roots and shoots was calculated using an electronic balance.

### 4.4. Photosynthetic Indicators

The plants were dark-adapted for 30 min, and then leaf Fv/Fm (maximum quantum efficiency of PSII photochemistry) and ETR (photosynthetic electron transfer rate) were measured using the PAM-2500 chlorophyll fluorometer (Walz, Effeltrich, Germany); Pn (net photosynthetic rate), GS (stomatal conductance), Ci (intercellular carbon dioxide), and Tr (transpiration rate) were measured using the LI-COR 6400 (USA) portable photosynthesis system to determine Pn, GS, Ci, Tr; the chlorophyll (Chl) content was determined spectrophotometrically after extracting with 95% ethanol [64]; the three-enchiral NR110 colorimeter was used to determine the front and back of the leaves of the purple flowering stalks, and the samples were aligned with the light collection hole of the colorimeter to take readings of the leaves. The L*, a* and b* values were recorded.

### 4.5. Antioxidant Capacity and Osmoregulation Indicators

SOD (superoxide dismutase) was determined via the nitrogen blue tetrazolium method [65], POD (peroxidase) was determined via the guaiacol method [66], CAT (catalase) was determined via the method of Patra et al. [67], APX (ascorbate peroxidase) was determined via the method of [68], and GR (glutathione reductase) activity was determined by a kit (BC1160 Solarbio, Beijing, China). Soluble proteins were determined via the method of Coomassie Brilliant Blue [69], soluble sugars were determined via the anthrone reagent method [70], proline was determined via the method of sulfosalicylic acid [71], and MDA was determined via the method of thiobarbituric acid [72].

### 4.6. Contents of ASA and GSH

The ascorbic acid content was estimated spectrophotometrically using the red phenanthroline method, utilizing the Evolution 300 spectrophotometer from Thermo Fisher Scientific, Waltham, MA, USA [73]. Furthermore, 0.5 g plant leaves were powdered in liquid nitrogen, and 1 mL of 2.5 M perchloric acid was added. The extract was centrifuged (2 °C, 10 min, 10,000× *g*). The supernatant was neutralized with saturated Na_2_CO_3_ using methyl orange as an indicator. Reduced ascorbate was spectrophotometrically measured at 265 nm in 1 M NaH_2_PO_4_ buffer (pH 5.6) with 1 U ascorbate oxidase.

GSH contents were assayed using the 2-nitrobenzoic acid (DTNB)-glutathione reductase (GR) recycling method, as described by Yu [74] with some modifications. Aliquots of the supernatant (0.4 mL) were neutralized with 0.5 M K-phosphate buffer (pH 7.0) (0.6 mL) prior to the assay.

### 4.7. Composition and Content of Glucosinolates

As previously mentioned [75], glucosesinolates were examined. High performance liquid chromatography (HPLC) was used to examine desulfoglucosinolates. The Agilent 1260 HPLC apparatus with a variable wavelength detector (VWD) was used for the analysis. Acetonitrile, water, and a Waters Spherisorb C18 column (250 × 4.6 mm) were used to separate the samples at a temperature of 30 °C and a flow rate of 1.0 mL/min^−1^. On a 250 × 4.6 mm Waters Spherisorb C18 column, the separation was carried out. At 226 nm, the absorption was found.

### 4.8. Cadmium Content

At harvest, roots and shoots were separated and dried at 105 °C for 30 min to fixation and then at 70 °C until the material reached a constant weight. The dried tissues were weighed and ground into a powder for the determination of Cd and mineral element concentrations, and the Cd content was determined using an atomic absorption meter (AA900T) after digestion with mixed acids [HNO_3_+HClO_4_ (3:1 *v*/*v*)] [76]. The translocation factor (TF) was defined as (Cd content in shoot)/(Cd content in root) [77].

### 4.9. RNA Sequencing and Analysis of Differentially Expressed Genes

On day 9, leaves of purple flowering stalks were treated with Cd and selenium. Regarding CK, CdCK, CdSe, and Se, samples from these four treatments were immediately frozen in liquid nitrogen and stored at −80 °C until RNA extraction. A total of 12 leaf samples were sent to Wuhan Seqhealth Technology Co. (Wuhan, China) for RNA extraction, library construction, and RNA sequencing. Reads containing articulators, reads containing poly-N, and low-quality reads were removed from the raw data to obtain clean data. High-quality paired-end reads from each library were mapped to the Brassica reference genome using TopHat v2.0.12 (http://brassicadb.cn/#/Download/ (accessed on 19 May 2023)), using Brassica rapa cv. Chiifu V3.0 as the reference genome. Genes differentially expressed between groups were identified using the edgeR package (version 3.12.1), which models the variability of raw count data of RNA-seq based on the negative binomial distribution [78,79]. Genes with |log2 Fold change| ≥ 1 and *p*-value < 0.05 were designated as differentially expressed. The NCBI SRA database has received our raw transcriptome data, which is associated with the BioProject accession number PRJNA1061651.

### 4.10. GO and KEGG Enrichment Analysis

The GOseq R package was utilized to conduct gene ontology (GO) enrichment analysis for DEG, with a correction for gene length bias (http://www.geneontology.org/ (accessed on 29 June 2023)). DEG determined that the GO keywords highly enriched were those with a corrected *p*-value < 0.05.A database for comprehending the sophisticated operations and uses of biological systems is called KEGG (Kyoto Encyclopedia of Genes and Genomes, Kyoto, Japan) (http://www.genome.jp/kegg/ (accessed on 30 June 2023)). In annotated single-gene sequences, we identified active biological pathways by mapping sequences to authorized pathways in KEGG.

### 4.11. Quantitative Real-Time PCR (qRT-PCR) Validation

qRT-PCR was carried out on the Bio-Rad iCycler thermocycler (Bio-Rad, Hercules, CA, USA) in accordance with the guidelines provided by the TB Green Premix Ex Taq II (Tli RNaseH Plus) kit. Every qRT-PCR reaction was conducted in three parallel reactions, with each biological repetition being independent. Table S4 contains a list of primers utilized in this investigation.

### 4.12. Statistical Analysis

Excel 2010 was used for statistical analysis of the experimental data, while SPSS 20.0 was used for ANOVA analysis. *p* < 0.05 was the significance level. The statistical software utilized was SPSS 17.0 (SPSS Inc., Chicago, IL, USA). The significance of the differences between treatments (*p* value < 0.05) was evaluated using Duncan’s test.

## 5. Conclusions

We discovered how exogenous selenium strengthened purple flowering stalks’ resistance mechanism to cadmium exposure by physiological and comparative transcriptome analysis. In the leaves of purple flowering stalks under Cd stress, exogenous selenium treatment increased the glutathione content, photosynthetic capacity, and antioxidant enzyme activities while decreasing MDA (malondialdehyde), proline, soluble protein, and the soluble sugar contents. Assays for regulatory genes involved in metabolic pathways including glucosinolates and differentially expressed genes (DEGs) linked to the selenium response under cadmium stress were also carried out. The Cd stress-responsive genes found during the application of exogenous selenium serve as excellent potential targets for future molecular breeding, and these findings further our understanding of how exogenous selenium increases Cd stress in purple flowering stalks.

## Figures and Tables

**Table 1:** Effects of selenium treatment on photosynthetic parameters of purple flowering stalks exposed to cadmium. L*, a*, b*. According to Duncan’s test, different letters indicate a statistically significant difference (*p* < 0.05).

Treatments	L*				a*				b*			
	0d	3d	6d	9d	0d	3d	6d	9d	0d	3d	6d	9d
CK	34.58± 2.28 a	42.55± 1.76 a	40.18± 1.54 a	35.86± 1.78 b	−14.64± 0.04 a	−13.8± 1.30 a	−13.34± 0.32 a	−13.64± 1.28 b	19.05± 0.13 a	16.92± 1.02 c	17.42± 1.49 c	12.50± 0.59 c
CdCK	34.58± 2.28 a	40.13± 1.35 a	42.35± 0.87 a	44.78± 2.27 a	−14.64± 0.04 a	−13.18± 1.04 a	−13.04± 0.63 a	−10.12± 0.47 a	19.05± 0.13 a	19.12± 0.15 b	22.56± 0.99 a	23.07± 1.86 a
CdSe	34.58± 2.28 a	42.66± 0.96 a	40.62± 2.5 a	38.61± 3.50 b	−14.64± 0.04 a	−14.52± 1.02 a	−13.97± 0.35 ab	−11.87± 1.18 b	19.05± 0.13 a	18.18± 1.25bc	19.57± 1.91 bc	16.18± 1.76 b
Se	34.58± 2.28 a	41.20± 0.91 a	41.66± 1.83 a	39.41± 2.97 b	−14.64± 0.04 a	−14.84± 0.42 a	−14.90± 0.63 b	−12.93± 0.40 b	19.05± 0.13 a	22.29± 0.50 a	20.48± 0.48 ab	17.00± 1.8 b

**Table 2:** Effects of selenium treatment on cadmium content of purple flowering stalks exposed to cadmium. ND: not detected. DM: dry matter. TF: translocation factor. According to Duncan’s test, different letters indicate a statistically significant difference (*p* < 0.05).

Treatment	Shoot Cd Content mg/kg DM				Root Cd Content mg/kg DM				TF			
	0d	3d	6d	9d	0d	3d	6d	9d	0d	3d	6d	9d
CK	ND	ND	ND	ND	ND	ND	ND	ND	ND	ND	ND	ND
CdCK	ND	116.3 a	132.6 a	294.7 a	ND	177.0 a	285.7 b	607.6 b	ND	0.66	0.47	0.49
CdSe	ND	59.3 b	35.6 b	135.1 b	ND	190.5 a	489.2 a	1933.4 a	ND	0.31	0.07	0.07
Se	ND	ND	ND	ND	ND	ND	ND	ND	ND	ND	ND	ND

## Data Availability

All data supporting the findings of this study are available within the paper and within its Supplementary Materials published online.

## Supplementary Materials

The supporting information can be downloaded at: https://www.mdpi.com/article/10.3390/ijms25031800/s1.
