# Supplementary material for: Transcriptome Analysis Reveals the Mechanism of Exogenous Selenium in Alleviating Cadmium Stress in Purple Flowering Stalks (Brassica campestris var. purpuraria)
Source: Int J Mol Sci. 2024 Feb 1;25(3):1800. doi: 10.3390/ijms25031800 (PMC10855379; doi:10.3390/ijms25031800)
Supplement: Supplementary file 1 [file ijms-25-01800-s001.zip › Table S1 Physiological parameters in Purple Flowering Stalks leaves with cadmium treatment and Se pretreatment.pdf]

Table S1 Physiological parameters in Purple Flowering Stalks leaves with cadmium treatment and Se pretreatment

Treatments	height (cm)	root length (cm)	Shoot fresh weight (g)	root fresh weight (g)	Fv/Fm	Y (II)	ETR	NPQ	Pn (μmol CO_2 m^{-2} s^{-1})	GS (μmol CO_2 m^{-2} s^{-1})	Ci (μmol CO_2 m^{-2} s^{-1})	Tr (μmol CO_2 m^{-2} s^{-1})	Chla content (mg/g FW)	Chlb content (mg/g FW)	Car content (mg/g FW)	total chl content (mg/g FW)	MDA (nmol /g)	SOD (U/g FW)	POD (U/g FW)	CAT (U/g FW)
CK	11.8±0.20 a	18.23±1.72 a	6.64±1.06 a	1.41±0.08 a	0.82±0.01 a	0.67±0.01 a	246.57±5.29 a	0.44±0.01 bc	20.78±0.81 a	0.46±0.01 a	281.75±3.86 c	3.35±0.0 a	9.61±0.64 a	3.58±0.23 a	0.77±0.08 a	13.19±0.87 a	10.72±0.96 c	147.29±12.32 a	570.87±76.39 a	1854.08±83.24 a
CdCK	3.83±0.25 e	6.30±0.69 d	0.88±0.17 d	0.27±0.04 d	0.33±0.14 b	0.12±0.02 d	37.60±9.96 c	0.75±0.09 a	3.35±0.38 e	0.17±0.01 c	355.74±6.85 a	2.01±0.05 c	1.85±0.12 c	1.10±0.04 c	0.06±0.06 c	2.95±0.16 d	20.07±0.76 a	63.07±15.58 d	298.50±31.43 d	874.94±90.14 d
T1(10 μmol/L)	4.57±0.15 d	9.43±0.55 c	1.45±0.30 cd	0.36±0.07 cd	0.34±0.07 b	0.22±0.03 d	47.80±11.80 c	0.52±0.08 b	6.77±0.36 d	0.19±0.02 c	313.25±8.32 b	2.14±0.35 c	2.16±0.42 c	1.22±0.26 c	0.16±0.02 bc	3.38±0.67 cd	15.06±0.24 b	99.66±5.14 c	387.50±54.49 cd	1279.1±104.08 c
T2(50 μmol/L)	5.53±0.35 c	11.77±1.00 c	2.37±0.27 bc	0.49±0.08 bc	0.77±0.02 a	0.37±0.07 c	188.27±20.28 b	0.43±0.08 bc	9.77±0.94 c	0.2±0.01 c	308.77±8.41 b	2.42±0.26 c	2.77±0.57 c	1.47±0.09 bc	0.21±0.07 b	4.24±0.54 c	9.49±1.57 c	124.18±10.69 b	462.50±66.14 bc	1508.26±140.49 b
T3(250μmol/L)	6.97±0.38 b	15.33±2.48 b	2.65±0.20 b	0.62±0.10 b	0.80±0.01 a	0.52±0.09 b	193.67±28.40 b	0.32±0.09 c	13.61±1.16 b	0.24±0.02 b	277.47±6.08 c	2.89±0.17 b	4.29±0.57 b	1.84±0.28 b	0.25±0.05 b	6.13±0.85 b	7.17±1.05 d	138.85±6.42 ab	555.00±26.46 ab	1833.27±113.42 a
