# Supplementary material for: Transcriptome Analysis Reveals the Mechanism of Exogenous Selenium in Alleviating Cadmium Stress in Purple Flowering Stalks (Brassica campestris var. purpuraria)
Source: Int J Mol Sci. 2024 Feb 1;25(3):1800. doi: 10.3390/ijms25031800 (PMC10855379; doi:10.3390/ijms25031800)
Supplement: Supplementary file 1 [file ijms-25-01800-s001.zip › Table S2.pdf]

Table S2. Effects of selenium treatment on photosynthetic parameters of Purple Flowering Stalks exposed to Cadmium. Chlorophyll a content, Chlorophyll b content, Carotenoid content.

Treatments	Chlorophyll a content (mg g^{-1} FW)				Chlorophyll b content (mg g^{-1} FW)				Carotenoid content (mg g^{-1} FW)				Total chlorophyll content (mg g^{-1} FW)			
	0d	3d	6d	9d	0d	3d	6d	9d	0d	3d	6d	9d	0d	3d	6d	9d
CK	2.84± 0.01 a	3.63± 0.2 b	5.49± 0.25 a	4.05± 0.32 a	1.62± 0.05 a	2.72± 0.15 ab	3.60± 0.08 a	2.89± 0.22 a	0.18± 0.02 a	0.38± 0.03 c	0.72± 0.06 a	0.53± 0.04 a	4.47± 0.04 a	6.34± 0.43 b	9.09± 0.33 a	6.94± 0.54 a
CdCK	2.84± 0.01 a	4.55± 0.33 a	3.66± 0.31 c	3.49± 0.10 a	1.62± 0.05 a	3.17± 0.35 a	2.53± 0.19 c	2.65± 0.18 a	0.18± 0.02 a	0.55± 0.03 b	0.38± 0.03 c	0.33± 0.02 b	4.47± 0.04 a	7.75± 0.78 a	6.19± 0.50 c	5.61± 0.33 b
CdSe	2.84± 0.01 a	3.48± 0.14 b	3.36± 0.30 c	3.65± 0.38 a	1.62± 0.05 a	2.45± 0.26 b	2.66± 0.30 bc	2.56± 0.23 a	0.18± 0.02 a	0.36± 0.03 c	0.51± 0.01 b	0.35± 0.01 b	4.47± 0.04 a	5.74± 0.18 b	6.25± 0.02 c	6.21± 0.60 ab
Se	2.84± 0.01 a	4.57± 0.24 a	4.42± 0.10 b	3.69± 0.29 a	1.62± 0.05 a	3.04± 0.15 a	2.98± 0.28 b	2.62± 0.15 a	0.18± 0.02 a	0.62± 0.01 a	0.56± 0.02 b	0.38± 0.03 b	4.47± 0.04 a	7.62± 0.39 a	7.71± 0.31 b	6.31± 0.44 ab
