# Supplementary material for: Transcriptome Analysis Reveals the Mechanism of Exogenous Selenium in Alleviating Cadmium Stress in Purple Flowering Stalks (Brassica campestris var. purpuraria)
Source: Int J Mol Sci. 2024 Feb 1;25(3):1800. doi: 10.3390/ijms25031800 (PMC10855379; doi:10.3390/ijms25031800)
Supplement: Supplementary file 1 [file ijms-25-01800-s001.zip › Table S3 Sequencing statistics of the transcriptome from four treatments of Purple Flowering Stalks.pdf]

Table S3 Sequencing statistics of the transcriptome from four treatments of Purple Flowering Stalks

Sample	Raw Reads	Raw Bases(G)	Raw Q30(%)	Raw GC(%)	Clean Reads	Clean Bases(G)	Clean Q30(%)	Clean GC(%)	Effective Rate(%)	total_reads	total_mapped(%)	unique(%)	read1(%)	read2(%)	reads_map_plus(%)	reads_map_minus(%)
CK-1	57241930	8.59	92.94	47.69	49791448	7.4	96.44	47.6	86.98	49791448	48196893(96.80)	46689150(96.87)	23344578(50.00)	23344572(50.00)	23344575(50.00)	23344575(50.00)
CK-2	64388976	9.66	94.38	47.79	57966918	8.57	97.01	47.68	90.03	57966918	56030441(96.66)	54286415(96.89)	27143210(50.00)	27143205(50.00)	27143209(50.00)	27143206(50.00)
CK-3	54470298	8.17	93.97	47.61	48526578	7.19	96.88	47.5	89.09	48526578	46864836(96.58)	45496778(97.08)	22748391(50.00)	22748387(50.00)	22748388(50.00)	22748390(50.00)
CdCK-1	56723202	8.51	94.18	47.62	50936414	7.55	96.84	47.52	89.8	50936414	49265813(96.72)	47838081(97.10)	23919041(50.00)	23919040(50.00)	23919041(50.00)	23919040(50.00)
CdCK-2	54854072	8.23	93.86	47.82	48851292	7.25	96.71	47.71	89.06	48851292	47150704(96.52)	45718498(96.96)	22859250(50.00)	22859248(50.00)	22859249(50.00)	22859249(50.00)
CdCK-3	50437904	7.57	93.51	47.8	44432372	6.6	96.73	47.7	88.09	44432372	43043120(96.87)	41690922(96.86)	20845462(50.00)	20845460(50.00)	20845460(50.00)	20845462(50.00)
CdSe-1	56447566	8.47	94.44	47.51	50936534	7.55	97	47.38	90.24	50936534	48885497(95.97)	47567217(97.30)	23783610(50.00)	23783607(50.00)	23783610(50.00)	23783607(50.00)
CdSe-2	59255326	8.89	93.94	47.48	52785916	7.84	96.83	47.38	89.08	52785916	50875655(96.38)	49135361(96.58)	24567681(50.00)	24567680(50.00)	24567681(50.00)	24567680(50.00)
CdSe-3	53958948	8.09	94.05	47.55	48261820	7.16	96.81	47.43	89.44	48261820	46410426(96.16)	44853662(96.65)	22426832(50.00)	22426830(50.00)	22426831(50.00)	22426831(50.00)
Se-1	53433380	8.02	93.81	47.67	47470096	7.05	96.78	47.58	88.84	47470096	45812114(96.51)	44250800(96.59)	22125401(50.00)	22125399(50.00)	22125401(50.00)	22125399(50.00)

Se-2	54373584	8.16	93.96	47.46	48552904	7.21	96.78	47.35	89.3	48552904	46437140 (95.64)	44577123(95.99)	22288564 (50.00)	22288559 (50.00)	22288561(50.00)	22288562(50.00)
Se-3	52040458	7.81	93.46	47.39	45862352	6.82	96.62	47.31	88.13	45862352	44065358 (96.08)	42805090(97.14)	21402546 (50.00)	21402544 (50.00)	21402544(50.00)	21402546(50.00)
