# Supplementary material for: Transcriptome Analysis Reveals the Mechanism of Exogenous Selenium in Alleviating Cadmium Stress in Purple Flowering Stalks (Brassica campestris var. purpuraria)
Source: Int J Mol Sci. 2024 Feb 1;25(3):1800. doi: 10.3390/ijms25031800 (PMC10855379; doi:10.3390/ijms25031800)
Supplement: Supplementary file 1 [file ijms-25-01800-s001.zip › Table S4 Primer sequences used in this study for qRT PCR.pdf]

Table S4 Primer sequences used in this study for qRT-PCR

Gene ID	Gene name	Forward primer sequence (5′-3′)	Reverse primer sequence (5′-3′)
BraA05g027600.3C	*BCAT4*	ATTATCGCTACAGGGTGTACTG	ATCTGCAACCTCCCGAATATAG
BraA06g012350.3C	*CYP79F1*	CAGTTTGGGGAGATTTTGGAAG	AGGTTGCAATTTGCTCTTCTTC
BraA04g029510.3C	*CYP83A1*	TCTTTTACATCTTCACCGTGGA	CTGTGTTTTCGCTAGCTTTCTT
BraA03g020140.3C	*WRKY33*	TTTGTTTGCTGAAGAAGTCACC	CTTCGGTGTCGTAATCAATCAC
BraA04g022100.3C	*GST*	TAGCTTTGGTGTTGTGCTTTAC	GCAAATATCAGCCAATTTTGCC
BraA09g021180.3C	*CYP707A*	ATCTGATGAATTCTTGAGGGCA	GAATTTGAGATGCCCTAACGTC
BraA01g013470.3C	*ANS*	ACGGCGATAACAAGAGAGTTAA	GAATAAGTCGAAGATGCGTTGG
BraA03g034380.3C	*sir*	TCGGATCGAATACTCTGACAAG	ATGAATAGCGTAATGGACCCAT
BraA08g009680.3C	*SELENBP1*	CGATCAATGCACTTACAGGAAG	CACAAGCAAAGGTTATGCAGAT
BraA01g004020.3C	*ALDH*	GATGTTCAACGGATTCTACAGC	ACAATGTGCTGGATCATAGTGA
BraA01g028620.3C	*petF*	ATACGCTACGTCAAGTTCTTGA	GAAATCCATGACGTCTTCATCG
BraA09g029800.3C	*ATPF1D*	AACCTGAGATCAAAGTGCCTTA	CACAGCAATGACACTTGTTGTA
BraA07g010120.3C	*psaF*	GAAGGATAAGAACGAGGACGAT	TGGATCTTTCTTGTCTTCAGGG
BraA07g031170.3C	*psbY*	CTACCTGGAAGCTGTTTCAGTA	CCTCCGAAAACACTTTTAACGT
BraA01g008860.3C	*psb28*	ATGGACCTGTACTGAACCAATT	AGCTCCAACAGAAACTCTACTC
BraA02g025510.3C	*WRKY40*	ACTTGAAGAATCTCGCTCTCTG	ATCAGGATCATGAGGAATGTCG
BraA07g038080.3C	*SOT16*	AATTTCAAGATTCTGCCCATCG	CGGTTGAATCCGTCTTTGTATC
BraA04g017740.3C	*CARP*	GATCCCTGAGTTGAAGCACTAT	TTGTGATTTTGAAGAGCACTCG
	braActin	CGGTGTCATGGTTGGGAGA	CGTGCTCGATGGGGTACTTC
