# Supplementary figures and images for: Transcriptome Analysis Reveals the Mechanism of Exogenous Selenium in Alleviating Cadmium Stress in Purple Flowering Stalks (Brassica campestris var. purpuraria)
Source: Int J Mol Sci. 2024 Feb 1;25(3):1800. doi: 10.3390/ijms25031800 (PMC10855379; doi:10.3390/ijms25031800)

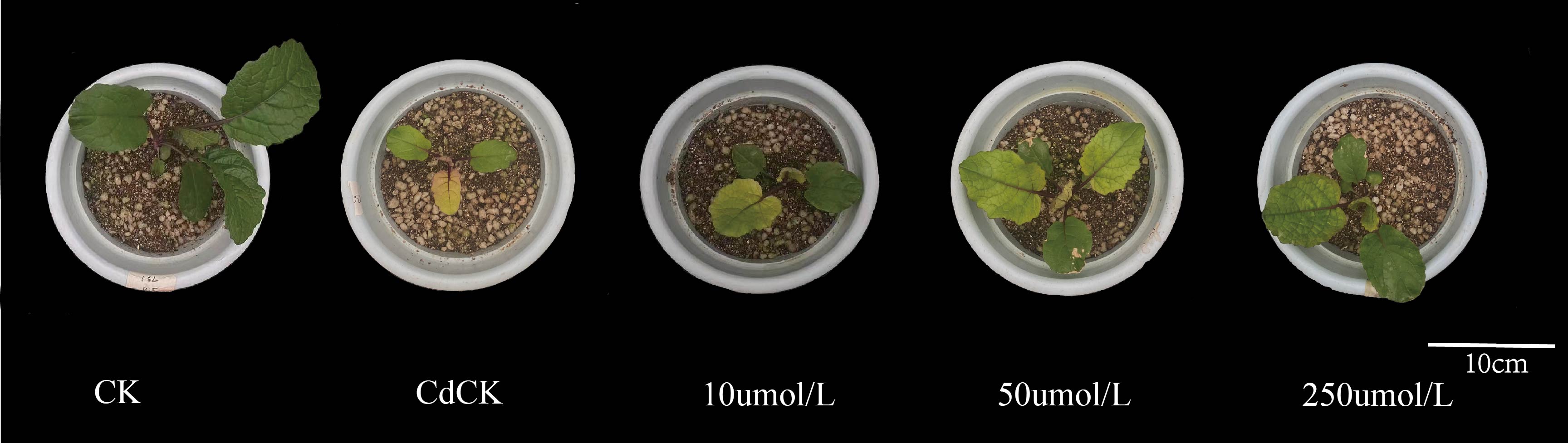

Supplement: Supplementary file 1 [file ijms-25-01800-s001.zip › Figure S1 Phenotype of Purple Flowering Stalks with different concentrations of selenium pretreatment after cadmium stress.jpg]

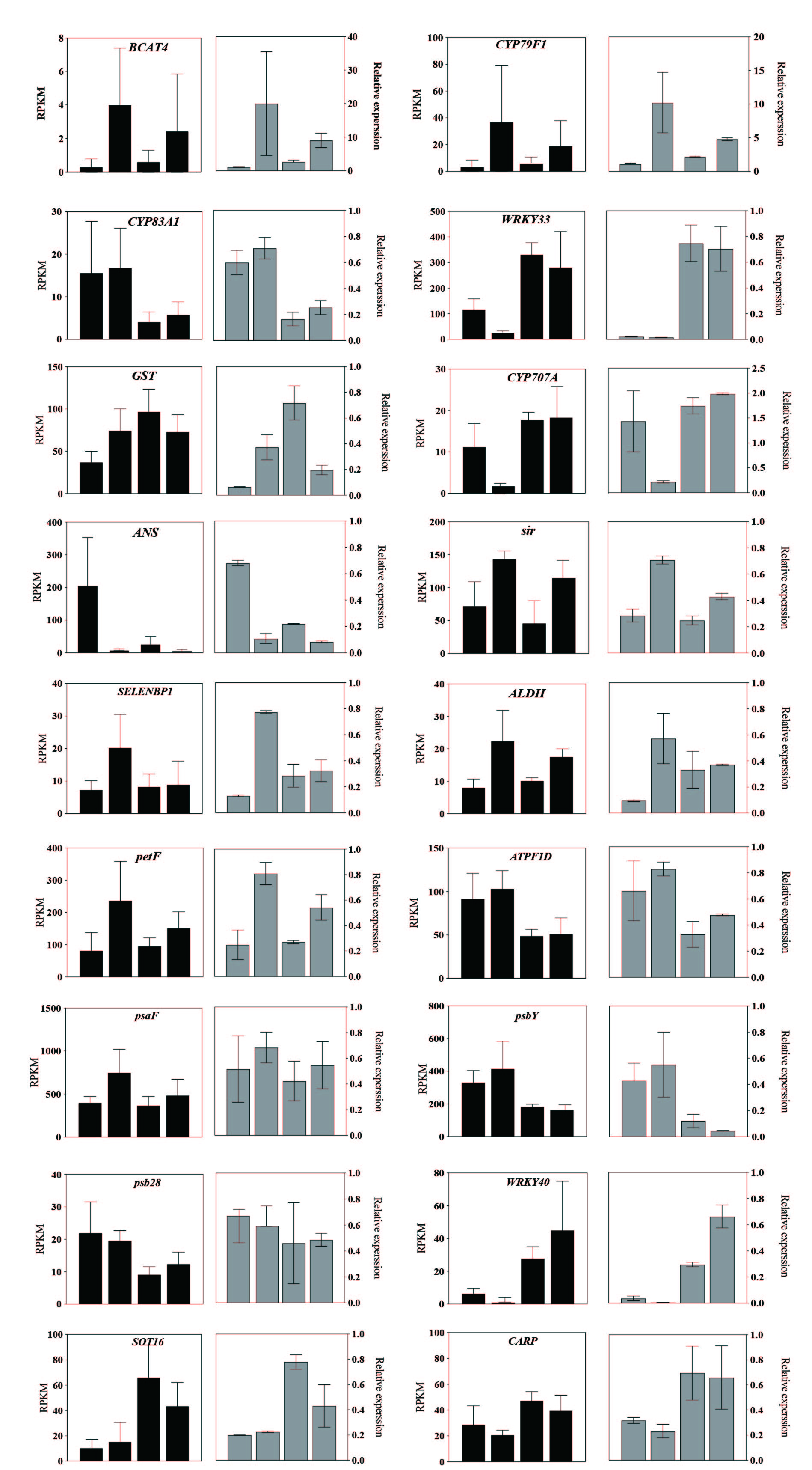

Supplement: Supplementary file 1 [file ijms-25-01800-s001.zip › Figure S2.Expression pattern of 18 selected DEGs obtained by qRT-PCR and RNA-seq.jpg]
